# Report of C. B. Coventry, M. D., on Epidemic Cholera

**Published:** 1848-08

**Authors:** 


*Report of C. B. Coventry, M. D., on Epidemic Cholera.* Addressed to the Faculty of the Medical Institution of Geneva College, and of the Medical Department of the University of Buffalo. July, 1848.

GENTLEMEN:—In accordance with my instructions, to "visit Europe for the purpose of investigating the causes, and ascertaining, as far as possible, the best mode of prevention, and treatment of the Asiatic Cholera," I sailed from New-York on the 12th day of January, 1848, reached Havre on the 7th of February, and proceeded the next day, to Paris. I remained at Paris between five and six weeks; went from thence to London, where I remained until the latter part of April, when my duties in the University of Buffalo requiring my return, I sailed for United States. In compliance, also with my instructions, I respectfully report the result of such investigations.

On reaching Europe, I found that the progress of the Cholera westward was temporarily arrested. The political disturbances in different parts of the continent rendered travelling difficult, if not dangerous, and all conspired to induce me to abandon the attempt to meet the disease. I, however regretted this necessity the less, when I learned that the symptoms and characters of the disease were substantially the same as when it prevailed in this country in 1832, at which time I had ample opportunity, both in the city of New-York, and Utica, of witnessing its symptoms. The object of my mission was not so much to see the disease, as to ascertain whether it differed materially from the Cholera as it prevailed in this country in 1832, and to learn the views of the profession in Europe as to the causes, the best means of prevention, the pathology, and the treatment of this formidable disease. I am aware that some enlightened members of our profession considered such a mission unnecessary, and boasted that they knew how to treat the disease as well as any Physicians in Europe. I certainly would be the last to question their capacity and skill. It should, however, be remembered, that the profession are not all equally enlightened. You, at least, thought that there was something still to be learned, and believing it a duty of the medical profession to neglect no means of guarding against the "Pestilence that walketh by day," you did not hesitate to act. If there be any truth in the following observations of the able editor of the Medico-Chirurgical Review, no reasonable person would question, as it seems to me, the propriety of such a mission:

"We may presume that there is not one of our readers whose attention is not fully awake to the probability—we might almost say, the certainty—of the return to our shores of that dreaded visitant, whose progress has never been checked in its course, or in any considerable degree averted by precautionary measures; the impenetrability of whose nature has hitherto defied the acumen of the most distinguished scientific investigation, and whose fearful results have left upon the minds, alike of the professions and the public, the lamentable impression of the utter incapacity of the resources of the therapeutic art to cope with its destructive energies."—*Med. Chir. Review, January,* 1848.

In addition to the opportunities of learning the views of the profession in Europe, I considered myself particularly fortunate in forming the acquaintance of Dr. Charles Searl, of Bath, England. Dr. Searl was long attached to the East India establishment at Madras, and during the prevalence of the Cholera in Europe in 1830, was physician-in-chief to the principal Cholera Hospital in Warsaw. Dr. S. published a work on the subject of the Cholera, some years since, and last season a new work on Cholera, Dysentery, and Fevers, combining the results of his vast experience and extended observation. Perhaps the highest compliment that could be paid to this work, is the fact that it has been furnished by the East India Company to all the medical officers in their employ, as the authrity by which they are to be governed. It was no small gratification to find that the views at which I had arrived, were substantially the same as those embraced by Dr. S.; and although there is much diversity of opinion, I believe these views are those of a large portion of the intelligent of the profession, and, so far as regards contagion, are concurred in, by the very intelligent board of Health Commissioners of England, who have discarded the idea of the cholera being contagious. How much of suffering, and of expenditure of money, would have been saved by a knowledge of this single fact in 1832!

*Causes of the Disease.*—The Cholera, as is well known, has existed in India, from time immemorial, but has prevailed sometimes as an endemic, at others as an epidemic, since 1817, varying in severity in different years, usually endemic, and apparently depending upon causes analogous to those which produce cholera morbus in our own country; viz: a high degree of temperature combined with malarious exhalations, and often excited into action by sudden vicissitudes of temperature, and irritating and unwholesome food. Occasionally the disease becomes much more virulent, is more extended, and assumes the form of an epidemic.

The question of greatest practical importance, and on which the profession are divided, relates to the contagious or infectious nature of the disease. They who remember the scenes of 1832, well know the amount of money expended in the establishment of quarantine regulations in every city and petty village, and their utter inutility in checking the progress of the disease. Speaking of the epidemic causes of the disease, Dr. Searl says:

"I may venture, without fear of contradiction, to say, that the influencing cause consists in some condition of the atmosphere which operates by reducing the active power of the system, and subverting the normal or healthy manifestations of life, and may, therefore, without impropriety, be called poisonous."

The ordinary causes, Dr. S. thinks, are similar in character and effect, though less active and virulent. They consist of the combined influences of the debility produced by long continued heat, the derangement of the biliary and associated organs resulting from a high temperature, and the poisonous influence of malaria, which is generated by the action of a tropical sun on vegetable and animal substances in process of decomposition. In these views I fully concur, but the limits of this report will not permit of our going into the evidences upon which such an opinion is founded.

The Metropolitan Sanitary Commissioners of England, in their Report presented on the 19th of February, 1848, say, "that the more recent experience in Russia has led to the general abandonment of the theory of its propagation by contagion, a conclusion in which, after a full consideration of the evidences presented to us, we fully concur." Although the idea that the cholera can be propagated by contagion in a healthy atmosphere is generally abandoned by the profession, there are many advocates for what is termed "contingent contagion," that is—that many diseases which are not communicable in a healthy atmosphere, may become contagious in a close, confined, or impure atmosphere. The number of believers in contingent contagion is increasing, and there is strong reason to believe that Cholera is one of the diseases to which the doctrine is applicable. Whilst the strongest evidence exists of its origin being independent of any communication with the sick, it is impossible to say that it may not be communicated in this manner in a contaminated atmosphere.

Whatever be the nature of the poison, and whether its primary action be on the blood, or the nervous system, the effect is evidently to prevent those changes in the circulation by which heat is generated, and the vital current purified. Carbonic acid is not formed by the union of the carbon with oxygen; heat is not generated; the blood, still charged with carbon, paralyzes the action of the heart, which, in its turn, becomes enfeebled; congestion of the internal organs follow, as in actual asphyxia; the nervous system being also paralyzed, the congested organs are incapable of retaining their contents, and the watery portion of the blood escaping by transudation into the intestinal canal, is ejected by vomiting and purging—hence, the discharge—hence, the discoloration from retention of the carbon—hence, the extreme cold, in consequence of heat not being generated, and of the rapid evaporation from the surface—hence, also, the dark colour, and thickened consistency of the circulating fluid, and, hence, too, the reason why injection of saline fluids into the veins, seldom succeeds in restoring the patient, though it may revive him for a time, for it cannot remove the congestion of the capillary system.

*Predisposing Causes of the Disease.*—If we are correct in our opinion that the exciting cause is some poisonous influence in the atmosphere, then it is evident that any, and every cause which tend to exhaust and depress the vital energies, must render the system more susceptible to the disease, whether this be exhaustion from over fatigue, from want of proper nourishment, from want of rest, from dissipation, or from fear. Few things have a more depressing effect upon the vital energies than fear; and it was a common observation in 1832, that many cases of cholera were produced by fright. Dr. E. A. Parker, of London, in a recent work on the subject, has advanced, and endeavored to maintain the doctrine, that the poison of the atmosphere acts primarily on the blood. I am, however, disposed to believe that the condition of the blood upon which he founds his principal evidence, is not the primary affection, but that the impression is primarily on the nervous system, prostrating its energies, and thus preventing those changes in the condition of the blood, and nutritive processes, by which the blood is purified, and caloric evolved. Dr. Parker thinks that the changes induced in the function of respiration, are directly consequent upon the alteration of the blood, and are the proper and distinctive sypmtoms of the disease. We suppose, on the contrary, that the changes in the function of respiration are induced by deficiency in nervous energy, and that the altered state of the blood follows as an effect, instead of being the cause. In the post mortem examinations, made by Dr. Parker, it was found that the blood coagulated very imperfectly, or not at all. This appeared to be owing to the diminution, or entire absence of fibrin. The blood was found accumulated in the internal organs, in the large vessels, and right side of the heart, being dark colored, and of thicker consistence than natural. The greater consistence must be attributed to the serous portion of the blood having been thrown off by the copious evacuations by the stomach and bowels.

*Symptoms.*—The Commissioners to whom I have already referred, state, "that the disease, as it has recently appeared in Persia, in Trebizond, and in Russia, is unchanged in its general character;" and that "it is at the present time, according to the latest information, in a similar position to that in which it was in 1831, when its progress was arrested by the frost, previous to its advance upon Europe immediately after the thaw took place." I shall not hesitate, therefore, in my account of the symptoms to describe those seen in this country in the epidemic of 1832. The first symptoms of Cholera vary very much, depending upon the intensity of the cause, the previous health and constitution of the patient, and the collateral and attendant circumstances. In most cases, the severer forms of the disease are preceded for a few days by more or less derangement of the stomach and bowels, loss of appetite, flatulence, and often diarrhœa. These symptoms may continue some two or three days, or may be present only as many hours. They are followed by frequent and profuse discharges from the bowels, not attended with pain, consisting, at first, of the ordinary contents of the bowels, but soon they become thin and watery, colorless, or of a light straw color, and hence not inaptly termed the rice water discharges. Nausea and sickness of the stomach, with vomiting, soon succeed, followed by cramps of the muscles of the extremities. To this succeed coldness of the surface and tongue; profuse clammy sweat; oppressed respiration, and the peculiar leaden hue of the countenance, which is characteristic of the advanced stage of the disease. In some of the more severe forms, the patient is suddenly seized with extreme prostration, vomiting, purging, and cramps, occurring almost simultaneously, and very rapidly succeeded by the algid state, or stage of collapse. In the most malignant form, the patient is suddenly prostrated, and expires, without passing through the several stages just described. The commissioners heretofore mentioned, consider the vomiting, active purging and cramp, as constituting the second stage of the disease; and what has usually been described as premonitory symptoms, viz. a slight diarrhœa, a sense of debility, flatulence, &c., as constituting the first stage.

*Pathology.*—The views expressed concerning the causes and symptoms of cholera, indicate our views of its pathology. As has been stated, we believe the first step in the morbid process is a depression of nervous energy, caused by the action of an impure air, which may act either from a deficient proportion of oxygen, or from containing an actual poison. As a consequence of this depressed nervous energy, those changes are not effected in the circulating fluid by which the carbon is eliminated; the heart's action is impaired, or lessened, by this vitiated condition of the blood; congestion of the internal organs, especially of the portal system and right side of the heart, follow as a consequence. The vomiting and purging, which are usually the most prominent symptoms, should properly be considered as efforts of the system to free, or relieve itself of the congested state of the internal organs. The profuse sweats arise from the relaxed condition of the capillary system, from want of nervous energy, and should not be confounded with a healthy and natural sweat resulting from increased activity of the capillaries of the skin. The extreme cold, results, partially, from evaporation from the surface, but mainly, from the absence of those changes by which animal heat is generated. In the more malignant forms of the disease, the patient either sinks from the sudden prostration of the nervous system, or dies exhausted by the profuseness of the discharges. In the milder forms, reaction takes place; the discharges are arrested, and if the congestion is not too great, the balance of the circulation is restored, and the patient recovers. If, however, the disease has progressed too far, inflammation of the congested organs supervene, and we have a febrile condition resembling typhus.

*Treatment.*—If the views which we have taken of the causes and pathology are correct, it is self evident that there can be no specific for cholera; that what may be proper in one stage of the disease, would be inappropriate in another;—in short, that, like other diseases, it must be treated on general principles. All persons acquainted with Cholera admit, that few diseases are more manageable, or yield more readily to treatment, in its first stage. In the second stage, or after vomiting and purging, with or without cramps, have commenced, the result is very doubtful, and in the third, or what is usually termed the stage of collapse, the case is all but hopeless. The first, and most important step, is to impress upon community the necessity of applying for advice in the first stage, or during what have usually been termed the premonitory symptoms. The latter term should be abolished, as both incorrect, and injurious in its effects. The Sanitary Commissioners of England are undoubtedly right in considering this the first stage of Cholera; and were patients told, and impressed with the belief that they then had the disease, they would more readily submit to the necessary treatment, than if merely told they had the premonitory symptoms. Much undeserved odium has been cast on the medical profession from their supposed inability to cure cholera; whereas it was, in fact, only the advanced stage of the disease, and when the system was no longer susceptible to the action of medicinal agents, that they failed to cure the disease.

In the first stage of the disease, the patient should confine himself to his bed, and take some mildly aromatic drink, such as an infusion of spearmint, or camomile, or warm camphor julep, until reaction takes place. The perspiration which follows should be encouraged by diluent drinks; this may also be promoted by a powder composed of three or four grains of Dover's powder, and one of calomel; or a pill composed of camphor gr. ss., opium and ipecac, aa. gr. one-fourth, calomel gr. i. repeated every two hours; after the sweating has continued three or four hours, the surface should be dried with warm flannel cloths, and a fresh and clean dress of flannel put on. When five or six of the pills have been taken, they should be suspended, and a small dose of rhubarb and magnesia, or of pure castor oil, should be given. Active, or drastic, or saline cathartics should not be given, and if castor oil is used, great care should be taken to see that it is pure and fresh. Most disastrous consequences have resulted from giving castor oil that was rancid. Mild nourishment should be given from time to time. If found necessary, the pills and cathartics may be repeated. The object should be to remove nervous prostration, and the congestion which has already commenced, by equalizing the circulation.

It is believed that this treatment is all that would be required in a large majority of cases, if seen in the first stage. Some modification is occasionally required. When there is nervous oppression at the stomach, without vomiting, a mild emetic will be found servicea le, and if the breathing is oppressed, the application of cups over the chest, and in come cases blood-letting may be required.

In the second stage of the disease the indications are the same, though modifications may be necessary in the mode of fulfilling them. If the patient is vomiting, he should be permitted to drink freely of some tepid drink. The spearmint tea, or weak chicken tea, may be used for this purpose. These should be continued though the patient continues to throw them off. Mustard cataplasms should be applied over the region of the stomach, and warmth to the surface. A good mode of applying warmth is by placing bags of heated salt along the limbs. The pills should be given as before, except that they should be more frequent, viz: every hour, or every half hour. We should not be deterred from giving the pills on account of the vomiting. If we succeed in co.recting the vomiting and purging, the cathartics should be administered as before; waiting, however, some ten or twelve hours. Bleeding from the arm, and the application of the cups, may in some cases be necessary, as before. In some cases, where an inflammatory condition of the stomach has supervened on the congestion, cold drinks will be preferred by the patient; in this he should be indulged. I have known small bits of ice, swallowed from time to time, directly useful, when it was found agreeable to the patient.

When the symptoms of cholera have been relieved, but the patient, instead of convalescing, falls into a typhoid state, it is an evidence that the congestion of the internal organs has not been fully relieved, and we must adopt such measures as the constitution of the patient will admit of; it may be V. S., counter irritation, or cupping over the region of the stomach, but I believe one of the most efficient means will be the use of calomel in small doses, so as to produce slight ptyalism.

In the third stage, or that of collapse, our chance of success is very slight, but the indications are the same as before. The first object must be to bring about a reaction; and for this purpose, in addition to the former means, the most powerful stimulants may be necessary to be employed. As soon as there are indications of reaction, bleeding may even be necessary in this stage of the disease, but not until reaction has commenced. It is impossible to point out all the modifications of treatment which the exigencies of the case may require, but they will readily suggest themselves to every intelligent practitioner. It may not, however, be improper to notice, very briefly, some of the remedies and means which have been used and recommended by different writers.

*Opium.*—Perhaps no article has been more used than opium. Looking only at two of the prominent symptoms, without adverting to the pathology, practitioners have given opium in large doses to check the purging and vomiting. The effect must have been to paralyze the nervous system, and increase the congestion. We are of opinion that opium can never be applicable in large doses in any stage of the disease. Given as recommended, in combination with calomel and ipecacuanha, it operates as a diaphoretic, and serves rather to excite, than prostrate the vital energies.

*Calomel.*—We have already expressed our opinion in favor of the administration of calomel, in small doses, with a view of exciting the secretions generally, and, under certain circumstances, carrying it to the extent of slight salivation. Doct. Searl considers calomel as a stimulant, exciting all the functions, including those of the heart and brain. He says, "that it does so, thirty years experience justifies me in asserting," and he adds, "it must of necessity (as it is a stimulant) act as an antagonistic agent also in suppression of the depressing influence of the cause of the disease; and if this be the case, it is a remedy, to which, under ordinary circumstances, we might apply the term specific in the cure of this disease; and, as the fruit of all my experience, I fearlessly aver, that it is as much so, as it is possible any single remedy can be." Doct. S. gives it in much larger doses than we have recommended. The objection to the large doses, as recommended by Doct. S., is, that it is liable to act as a cathartic, and thus defeat its own object. In the worst form of the disease, all power of absorption is lost, and, as has been observed, "medicines taken into the stomach have no more effect than if they had been put in the coat-pocket of the individual."

*Blood-Letting.*—Much diversity of opinion exists as to the utility of this remedy. Doct. Searl says, that if "not always indispensable, it is clearly a remedy, when judiciously employed, from which much benefit may frequently be derived, and which general experience has proved to be the case." When in New-York in 1832, I was permitted, through the kindness of a medical friend, to try the effect of bleeding on a patient whom he had given up as hopeless. I tied up the arm, and made a free opening into a large vein, but, with all my efforts at rubbing the arm, I got scarcely an ounce of blood, and that very thick and dark colored. This patient subsequently aborted, flowed freely, and recovered. In the milder forms of the disease, and particularly in the first stage, I believe blood-letting unnecessary; and in the severer forms, where the fluids of the system have been already drained off, that it would be inapplicable. On the other hand where evidence of local congestion continues after reaction has taken place, bleeding should be resorted to, the blood should be taken with the patient in a recumbent position, and the effect on the circulation carefully watched. If the pulse becomes fuller and stronger, the blood should be permitted to flow, but, if not, should be immediately stopped.

*Emetics* have been highly recommended by several members of the profession, both in India, and Europe. No one can doubt the powerful influence of vomiting in equalizing the circulation. It is the mode instituted by nature herself. But I believe that in the first stage they are usually unnecessary, and when the patient is already vomiting, I can see no advantage from their administration. In appropriate cases they are useful, but I must object to the administration of tart. emetic, and particularly in two grain doses, as recommended by some writers.

*Saline Injections* into the rectum, are strongly recommended by Doct. Searl. He says, "A large tea spoonfull of table salt dissolved in a pint of warm gruel, or water, and used as a clyster every half hour, the patient continuing in the recumbent posture, and passing it afterwards into a cloth, cannot be too strongly advised, and especially so, when vomiting is frequent, or spasms severe."

*Nutritive Injections* are strongly recommended by Doct. Searl, as a means of sustaining the system when every thing is ejected from the stomach.

*Saline Injections into the Veins* were, at one time, very highly extolled, during the former epidemic. Although the effect for a time was almost miraculous, patients often arousing from a state of insensibility, yet, unfortunately, this excitement was only temporary, the patient soon relapsing into his former condition. The operation is a difficult, and delicate one, and not without danger. Patients have occasionally recovered after such injections. It certainly would be improper to resort to injections into the veins, except in those cases which were hopeless from ordinary treatment.

*Inhalation of Oxygen Gas, and the Nitrous Oxide.*—I am not aware that the inhalation of Oxygen has been tried in Cholera, though strongly recommended by Doct. Searl, and some others. It would certainly be worth a trial in the severe forms of the disease. It is possible that we may find in this powerful agent a stimulant that would raise the system when other stimulants failed. The slight increase of oxygen produced by the combustion of nitre in the apartment, will sometimes relieve the suffering from asthma, and may, perhaps, prove beneficial in cholera.

*Means of Prevention.*—Whatever diversity of opinions may exist as to he contagious or non-contagious character of the disease, I believe that the profession, both in Europe, and this country, are nearly unanimous in the opinion that the quarantine regulations established in 1830–32, did not, in a single instance, prevent the occurrence, or limit the extent of the disease. On the contrary, whilst attended with great expense, they increased the panic, often prevented the possibility of procuring proper attendance on the sick, and caused the hurried, improper, and indecent interment of those who died, or were supposed to be dead. Dr. Searl says, that in the Cholera hospital at Warsaw, whilst under his charge, there was an average of from 30 to 60 cases under treatment; of this number half a dozen were buried daily. "Of thirty, or more attendants during three months that I was in charge, we had among this number only two cases of the disease, and the cause of the attack, in both cases was most satisfactorily explained. One of these men was not employed in attendance upon the sick, but in the kitchen preparing the food, and daily frequenting the shambles; the other, an hospital attendant, whom the apothecary finding intoxicated, had loockd up during the night in a damp cellar, with no covering but his shirt." A commission was sent by the French Government, who visited the hospital under the charge of Dr. S., and who tried a variety of experiments on animals, and on themselves, by taking the breath, and by inoculating themselves with the blood and excretions of the patients, without producing the disease. Dr. S. also mentions the fact of a gentleman having died of Cholera in his bed, and his occupying the same room, the same bed and clothing, the following night, without getting the disease.

Dr. Searl adds: "To these facts I may add those of daily occurrence in India, the disease attacking exclusively the men occupying the lower floor of a barrack, while those of the upper floor escaped; of its attacking the men sleeping on one side of a ship's deck in the roads, off Madras, only, or one portion of a cantonment, or the inhabitants of one bank of a river exclusively; or of the disease attacking a regiment on its march most virulently to day, and ceasing on the regiment's moving a few miles on the morrow." Another circumstance mentioned by Dr. S. as militating against the doctrine of contagion is, that, so far as we know, in all contagious diseases, the poison is generated by a febrile action of the system, whilst, in Cholera, there is no such febrile action. Dr. Parker, whom we have before quoted, "has never observed any indication of contagion in Cholera, all the phenomena of propagation, and development of the virus, which have fallen under his own observation, being sufficiently accounted for, without calling in the aid of the hypothesis that the virus can multiply itself by its action on the living human system." In the Medico-Chir. Review for January, 1848, we find the following observations:—"A general review of the whole case, then, leads us to this conclusion, that where the epidemic influence is strongly developed, infection is not likely to have any perceptible influence in propagating it; that the general march of the disease cannot be dependent on human communication, and that quarantine regulations, and similar restrictions upon intercourse, are utterly incapable of checking its progress; and that if human communication be in any case the immediate agency in its transmission, it can only be so when a strong predisposition has been occasioned by epidemic influence being dependent upon those health-destroying conditions which it is the object of the sanatary reform to remove." Dr. Milroy quotes, as the motto to his pamphlet on the Cholera, the following emphatic declaration of Mr. Farr in the report of the Registrar General:—"Internal sanatary arrangements, and not quarantine or sanatary lines, are the safe guards of nations against epidemic diseases."

Dr. Milroy says, "As surely has Cholera always sought out, and settled down upon the abodes of misery and filth, in every city of Europe, that has been visited by it, as the vulture-crows in the East, ever congregate where there is the most offal and garbage to be found." I will not go so far as to assert, that in an impure atmosphere, where a strong predisposition already exists, it may not be excited into action, by intercourse with the disease. This is a question which we have no means of determining. During the prevalence of the Cholera in 1832, I visited the several Hospitals in New York, which were filled with patients, in every stage of the disease; visited patients in the same city, in the worst abodes of poverty, wretchedness and filth; and during its prevalence in Utica, I was constantly engaged in attendance upon the sick, often sleeping in an apartment adjoining that of my patient, without contracting the disease. I am fully satisfied that many of the regulations adopted in 1832, although adopted with the best of motives, did more harm than good. No human agency has yet been found sufficient to arrest the progress, or avert the attack of the destroyer. But although we cannot prevent, much may be done to mitigate the severity of such a visitation. This is to be done by a careful removal, as far as possible, of every source of impurity of the atmosphere, and obviating all the ordinary causes of disease, by enjoining on the people the importance of strict temperance in eating, as well as drinking—the avoidance of everything that has a tendency to enervate or exhaust the system, and particularly the avoidance of drastic cathartics—in short, the strict observance of the Laws of Health. The medical profession should urge upon community, the importance of attending upon the first premonition of the disease. The first indication of derangement of the stomach or bowels, should be sufficient to confine the patient to his bed, and to send for medical advice. It is the universal testimony of the profession, that the disease usually yields readily to treatment in the first stage. The establishment of Cholera Hospitals, with the exception of particular circumstances, is worse than useless. Patients will not resort to them in the first stage of the disease, and their removal in the second stage, would be hazardous, if not fatal. The Sanatary Commissioners of England, say: "That the views which we have adopted in relation to the inexpediency of special Cholera Hospitals, except in cases of peculiar necessity, have been confirmed by the coincident adoption of the same conclusions in Russia." Should this scourge again visit our State, (which there is much reason to fear) special Cholera Hospitals would be necessary at all the cities and large villages on the line of internal navigation; not for the accommodation of the citizens, but for the prompt removal of persons sickening on boats, who would not find accommodation in private dwellings. A sufficient number of medical attendants should be engaged, at the public charge, to render prompt assistance in all cases where such attention may be required, and the corpse of no person, dying from Cholera, should be interred, until permission was granted, either by the public authorities, or the physician in attendance. There are few diseases where the danger of premature interment is so great as in cholera. The sudden prostration of nervous energy, as well as the sudden and profuse discharge of the fluids of the system, must frequently produce a state of syncope, which, in a time of general pain and alarm, might readily be mistaken for death, by an incautious observer. Fortunately, no necessity exists for a hurried interment. No poison has been generated by febrile action, which might contaminate the surrounding air, and thus prove injurious to others; decomposition is even slower in taking place than in deaths from ordinary causes. In no case should the body be interred in less than twenty-four hours after death.

I cannot consent to close my communication, without improving the opportunity of expressing my obligations, for the many marks of attention which I everywhere received from the members of our profession. I could not but feel proud of belonging to a profession that acknowledges no distinction of country, of language, or of creed, but whose members, when known, are everywhere received by members of the same profession as brethren.

I would more especially express my obligations to Dr. Pritchard, of London; Dr. Carpenter, Mr. Wm. Lawrence, and Samuel Cooper, of London, and Dr. Charles Searl, of Bath, England.

UTICA, July, 1848.

